# Sulfonamide Allergy and Alternative Treatments in Ocular Toxoplasmosis

**DOI:** 10.22336/rjo.2025.25

**Published:** 2025

**Authors:** Francisco Calleja Casado, Antonio Duch Samper

**Affiliations:** 1 Ophthalmology Service, Hospital Clínico Universitario de Valencia, Valencia, Spain; 2 University of Valencia, Valencia, Spain

**Keywords:** sulfonamide allergy, toxoplasmosis, ocular infection, alternative treatment, antibiotic therapy, uveitis, retinochoroiditis, immunology, ophthalmology, CNV = Choroidal Neovascularization, FA = Fluorescein Angiography, GWC = Goldmann-Witmer Coefficient, ICGA = Indocyanine Green Angiography, IOP = Intraocular Pressure, OCT = Optical Coherence Tomography, OCTA = Optical Coherence Tomography Angiography, PORT = Punctate Outer Retinal Toxoplasmosis, PCR = Polymerase Chain Reaction, RPE = Retinal Pigment Epithelium, TMP-SMX = Trimethoprim-Sulfamethoxazole, VEGF = Vascular Endothelial Growth Factor

## Abstract

**Purpose:**

To summarize current knowledge on the clinical features, diagnosis, and treatment of ocular toxoplasmosis, with emphasis on alternatives for sulfonamide-allergic patients.

**Materials and methods:**

A comprehensive PubMed search (1908-2021) was conducted using terms like *Toxoplasma gondii*, ocular toxoplasmosis, posterior uveitis, and retinochoroiditis. Priority was given to studies published after 2000, particularly those from 2010 onwards. Google Scholar, as well as English and Spanish sources, were also utilized.

**Results:**

Ocular toxoplasmosis is the most common infectious form of posterior uveitis worldwide, typically presenting as necrotizing retinochoroiditis with associated vitreomacular adhesion and vitritis. Diagnosis is mainly clinical, supported by serology (IgG/IgM) and PCR of intraocular fluids when needed. Standard treatment includes systemic antiparasitic agents (e.g., pyrimethamine with sulfadiazine or trimethoprim-sulfamethoxazole) plus corticosteroids. In patients with sulfonamide allergies, alternatives such as pyrimethamine with clindamycin or azithromycin, or intravitreal clindamycin with dexamethasone, are effective. Recent studies show comparable outcomes with these regimens. Prompt treatment of active disease helps reduce retinal damage.

**Discussion:**

Recent evidence supports the efficacy and safety of non-sulfonamide regimens and intravitreal therapies in managing ocular toxoplasmosis. These options are particularly valuable for patients with contraindications to standard treatments. Advances in imaging and molecular diagnostics have also enhanced early recognition and tailored management of the disease, contributing to improved visual outcomes.

**Conclusion:**

Advances in diagnostic tools (PCR, intraocular antibody detection) and alternative therapies have improved outcomes in ocular toxoplasmosis, including in patients who are intolerant to sulfonamides. Clindamycin-based or intravitreal approaches offer effective, safe options. Further research is needed to refine treatment protocols, prevent recurrences, and clarify disease pathogenesis.

## Introduction

Ocular toxoplasmosis, an infection of the retina and choroid by the protozoan *Toxoplasma gondii*, is the leading cause of infectious posterior uveitis in many populations [1,2]. Most infections with *T. gondii* are asymptomatic; however, a subset of individuals develop ocular involvement, which can result in significant vision loss. Classically, ocular toxoplasmosis presents as a focal necrotizing retinochoroiditis with a dense vitritis (“headlight in the fog” appearance), often unilateral in immunocompetent patients [3]. Both congenital infection and postnatally acquired infection can lead to ocular disease; current evidence suggests that most adult ocular toxoplasmosis cases are due to acquired infection rather than congenital reactivation.

In immunosuppressed patients or infants with congenital toxoplasmosis, the disease may be more severe, sometimes leading to complications such as retinal necrosis, glaucoma, or even blindness.

Prompt recognition of characteristic ocular lesions, coupled with confirmatory laboratory testing, is critical for accurate diagnosis and timely treatment. This review provides an overview of the clinical manifestations, diagnosis, and management of ocular toxoplasmosis, discussing therapeutic alternatives for patients with sulfonamide antibiotic allergies, who require special consideration in treatment planning.

## Materials and methods

A literature review was conducted to gather relevant information on ocular toxoplasmosis. PubMed was searched for articles up to 2021 using keywords including “*Toxoplasma gondii*”, “ocular toxoplasmosis”, “posterior uveitis”, and “retinochoroiditis”. Priority was given to research published in the last two decades, as well as classic references on the topic. Additional sources were obtained via Google Scholar to ensure comprehensive coverage. The search was restricted to English-language publications. Relevant data from clinical studies, reviews, and case series were extracted regarding epidemiology, pathophysiology, clinical features, diagnostic techniques, and treatments. Literature addressing management in the context of sulfonamide drug allergy was highlighted.

### Life cycle and epidemiology of toxoplasma infection

*Toxoplasma gondii* is an obligate intracellular coccidian parasite in the phylum Apicomplexa. The parasite exists in multiple forms: oocysts (shed in feline definitive hosts), rapidly dividing tachyzoites, and quiescent bradyzoites within tissue cysts. Cats excrete oocysts that become infectious 1-5 days after shedding; intermediate hosts (including humans) typically acquire infection by ingesting sporulated oocysts from contaminated soil or water, or by consuming undercooked meat containing tissue cysts [4]. Other routes include transplacental (congenital) transmission during acute maternal infection, organ transplantation, and rarely, blood transfusion. After ingestion, *T. gondii* transforms into tachyzoites that disseminate hematogenously. The parasite invades host cells and proliferates, causing cell lysis and local inflammation [4]. In immunocompetent hosts, a robust immune response usually controls the acute infection within a few weeks, driving the parasite into the latent bradyzoite stage. Encysted bradyzoites can persist for the lifetime of the host, predominantly in neural and muscular tissues, including the retina. Reactivation of cysts (release of tachyzoites) in the retina is thought to trigger the recurrent lesions characteristic of ocular toxoplasmosis.

### Epidemiology

*T. gondii* infection is widespread, but seroprevalence varies significantly by geography, ranging from ~10-30% in many temperate regions to over 60% in parts of Latin America, Continental Europe, and Africa [1,2]. This variability is influenced by climate (oocysts survive better in warm, humid environments), local dietary habits (such as the consumption of undercooked meat or unpasteurized milk), and sanitation practices. A substantial decline in seroprevalence has been observed in some high-income countries over the past few decades, likely due to improved hygiene and farming practices [1]. However, the trend could reverse with changes in animal husbandry and climate. Notably, outbreaks of toxoplasmosis have been linked to contaminated water supplies and other point sources; one large outbreak in India affected 248 people with acute acquired toxoplasmosis [3]. Environmental factors such as heavy rainfall and flooding can disseminate oocysts, leading to community outbreaks. T. gondii strain types also vary by region and may affect disease severity. In North America and Europe, type II strains are most common and often associated with milder disease in immunocompetent individuals. In contrast, in South America, more virulent atypical strains are prevalent and have been associated with more severe ocular manifestations. Global climate change has the potential to increase the transmission of *T. gondii* by expanding the range of felid hosts and enhancing oocyst survival [5].

### Pathophysiology of ocular toxoplasmosis

After entering the host, *T. gondii* tachyzoites disseminate and can invade the eye via the bloodstream. The parasite crosses the blood-retinal barrier likely by hitchhiking inside infected leukocytes (the “Trojan horse” mechanism) or by directly infecting vascular endothelium. Once in retinal or choroidal tissue, tachyzoites elicit a vigorous immune response. A combination of direct parasite-induced cell lysis and immune-mediated tissue damage leads to the necrotizing retinitis characteristic of ocular toxoplasmosis. The host immune response is primarily T-cell mediated, with interferon gamma (IFN-γ) and interleukin-12 (IL-12) playing a critical role in activating microglia and macrophages to control *T. gondii*. At the same time, the parasite has evolved mechanisms to modulate immunity: it injects effector proteins (e.g., ROP and GRA proteins) into host cells that can downregulate a strong Th1 response, allowing the parasite to persist in cyst form [6].

There is evidence that *T. gondii* triggers a complex immunologic cascade in the eye. Th17 cells and their cytokine IL-17 appear to play a dual role. IL-17A is a pro-inflammatory cytokine implicated in tissue damage; elevated levels of IL-17 have been detected in active toxoplasmic retinitis [7]. Paradoxically, IL-17 also has neuroprotective effects in specific contexts, helping to maintain immune privilege in the eye [7,8]. The net effect of Th17 activation in ocular toxoplasmosis is not fully understood; it may contribute to the inflammation necessary to control the infection, but also to bystander retinal damage. The severity of retinal lesions is determined by a delicate balance between parasite virulence factors and host immune responses.

Both host and parasite factors influence disease outcomes. More virulent parasite genotypes (such as type I strains) tend to cause larger, more aggressive retinal lesions, especially in regions where these strains dominate. Host immune status is equally important: in immunosuppressed patients (e.g., those with AIDS or on immunomodulatory therapy), even less-virulent strains can cause fulminant retinal necrosis due to inadequate immune containment. Other host factors, including age and genetic background, can affect susceptibility to severe ocular toxoplasmosis. For example, older patients may experience more granulomatous inflammation and are at higher risk of vision-threatening complications such as ocular hypertension and secondary glaucoma. In summary, ocular toxoplasmosis results from an interplay of the parasite’s propensity to establish persistent infection and the host’s immune response, which together determine whether a *Toxoplasma* infection remains latent or manifests as destructive uveitis.

### Clinical presentation and features

#### Symptoms

Patients with ocular toxoplasmosis most commonly report floaters and blurred vision in one eye, corresponding to the vitritis and retinal lesions. The severity of visual disturbance depends on the location of the lesion: lesions in or near the macula or optic disc often cause significant loss of visual acuity or scotomas, whereas small peripheral lesions may be asymptomatic or cause only mild visual field defects [9]. Photophobia and redness can occur if there is concomitant anterior uveitis. Pain is not a prominent feature unless intraocular pressure rises due to inflammatory glaucoma. In individuals with congenital toxoplasmosis, primary ocular toxoplasmosis may present in childhood or adolescence when a previously unrecognized retinal scar reactivates. These patients (or their parents) may notice a sudden drop in vision or the appearance of new visual floaters. In immunocompetent adults with acquired infection, ocular symptoms typically appear weeks to months after systemic exposure and often without notable systemic illness. By contrast, immunocompromised patients (e.g., HIV with low CD4 counts) can develop atypically severe, multifocal retinal lesions with more rapid progression; their symptoms may worsen over days rather than weeks.

#### Signs

On ophthalmic examination, active ocular toxoplasmosis typically presents as a focus of creamy-white necrotizing retinitis adjacent to an older, pigmented scar, accompanied by intense vitritis. The overlying vitreous haze often blurs the lesion margins. A moderate to severe vitreous cellular reaction gives the appearance of a “headlight in the fog”, where the white retinal lesion is observed through dense vitreal opacities [3]. Retinal vasculitis is common around the lesion - both arteries and veins can be sheathed with inflammatory exudates. In some cases, small yellow-white plaques may be observed along arterioles (Kyrieleis arteriitis), representing focal immune complex deposition on vessel walls. An associated anterior uveitis (iritis) is present in many patients, ranging from mild to pronounced; granulomatous keratic precipitates on the cornea and posterior synechiae (iris adhesions) can occur in prolonged cases. In immunocompetent individuals, ocular toxoplasmosis lesions are often solitary. Notably, more than 70% of patients presenting with an active lesion will also have at least one atrophic chorioretinal scar from a previous episode, indicating recurrent disease [1]. In immunosuppressed patients, multiple lesions in one or both eyes may be observed, and the retinitis can be multifocal or diffuse.

Most active lesions heal over 4-6 weeks, even without therapy, leaving a well-demarcated atrophic scar with hyperpigmented borders. The healed scar is usually smaller than the original lesion due to resorption of necrotic tissue. Punctate outer retinal toxoplasmosis (PORT) is an atypical manifestation characterized by numerous small, gray-white lesions at the level of the outer retina/RPE, with minimal vitritis. This form is thought to result from a different host-parasite interaction and can be mistaken for the white-dot syndromes. PORT often affects younger patients (many in their teens or twenties) and can be bilateral in about one-third of cases [9,10]. It may be associated with concurrent typical toxoplasmosis lesions in the same or fellow eye. Neuroretinitis (inflammation of the optic nerve with a macular star exudate) is a rare presentation of ocular toxoplasmosis. A few cases have been reported in which patients developed optic disc edema and a star-shaped pattern of hard exudates in the macula, mimicking the appearance of cat-scratch disease. Toxoplasmic neuroretinitis is usually accompanied by evidence of adjacent active retinitis, which helps suggest the correct diagnosis [11].

Scleritis is an uncommon manifestation in toxoplasmosis, but has been described, particularly in HIV-positive individuals with severe retinal inflammation. It is presumed that a full-thickness retinal lesion can extend inflammation into the choroid and sclera, causing a focal posterior scleritis [12].

#### Complications

Several complications can arise from ocular toxoplasmosis, especially with repeated inflammation or delayed treatment. Elevated intraocular pressure (IOP) occurs in a substantial fraction of cases due to inflammatory cells and debris blocking the trabecular meshwork (secondary glaucoma). This can cause ocular pain and further threaten vision if not managed. Cataract can develop from chronic anterior uveitis or prolonged corticosteroid use.

In the posterior segment, persistent vitritis and inflammation can lead to the formation of vitreomacular adhesions and epiretinal membranes on the retinal surface. Epiretinal membranes may cause distortion of the macula and can reduce vision even after the active retinitis has resolved; surgical peeling of these membranes can improve vision in select cases once the infection is inactive. Severe or recurring retinitis near the macula can result in full-thickness macular holes due to tissue loss and traction, as has been reported in long-term disease. In addition, chronic chorioretinal scar formation and disruption of Bruch’s membrane can promote choroidal neovascularization adjacent to old toxoplasmosis scars.

Patients with choroidal neovascular membranes may experience metamorphopsia (distorted vision) or a secondary drop in vision; treatment with intravitreal anti-vascular endothelial growth factor (anti-VEGF) agents often leads to regression of these neovascular membranes and stabilization of vision [13]. Rhegmatogenous retinal detachment is a less common but serious complication, usually arising when extensive necrotic retinitis in an immunocompromised patient leads to retinal tears. Serous retinal detachment can accompany active retinochoroiditis, particularly when subretinal fluid accumulates, especially in lesions near the macula. This typically resolves as the inflammation subsides, but if an active lesion is close to the fovea, even a shallow detachment can cause significant temporary vision loss. Finally, recurrent inflammation around the optic disc can result in chronic optic atrophy. Each additional recurrence of toxoplasmic retinochoroiditis carries a risk of cumulative retinal damage; therefore, preventing recurrences (e.g., through maintenance therapy in select cases) is a crucial aspect of long-term management.

#### Diagnostic work-up

The diagnosis of ocular toxoplasmosis is primarily based on clinical findings. A classic presentation in an otherwise healthy young patient - unilateral focal retinochoroiditis with dense vitritis and an adjacent old scar - is virtually pathognomonic. In such cases, laboratory confirmation, while supportive, is not always required before initiating therapy. However, atypical presentations or cases in immunocompromised hosts often necessitate ancillary testing to differentiate toxoplasmosis from other causes of necrotizing retinitis. The diagnostic work-up may involve both serologic tests and analysis of intraocular fluids.

#### Serologic testing

Serology establishes whether the patient has been infected with *T. gondii*. The presence of *Toxoplasma*-specific IgG antibodies in serum indicates past exposure (which is expected in most adults in endemic areas). A negative IgG effectively rules out toxoplasmosis as the cause of uveitis in an immunocompetent patient [2]. IgM antibodies indicate a recent or active infection; however, IgM can persist for months to years after an acute infection. Therefore, a positive IgM result is not definitive proof that ocular lesions are due to an active toxoplasmosis. In typical cases, most immunocompetent patients with ocular toxoplasmosis have positive IgG and negative IgM, reflecting an infection that was acquired at least several months earlier [14]. High IgG titers (or rising titers on paired samples) can support the diagnosis when clinical signs are suggestive [14]. For pregnant women or infants with congenital infections, specialized serologic assays, such as IgG avidity testing, are used to determine the timing of infection. A high IgG avidity (strong binding of IgG antibodies) in early pregnancy, for example, indicates that infection likely occurred more than 3-5 months prior, effectively excluding a recent primary infection that could endanger the fetus. In contrast, low IgG avidity in the presence of IgM suggests a recent infection. In congenital toxoplasmosis, infants may have circulating IgM, IgA, or persistent IgG beyond the disappearance of maternal IgG (which usually wanes by 12-18 months) [14].

A variety of serologic assays are used in the diagnosis of toxoplasmosis. Enzyme-linked immunosorbent assays (ELISAs) for IgG and IgM are commonly used due to their high sensitivity and automation capabilities. The Sabin-Feldman dye test, a historical reference assay that utilizes live tachyzoites, is highly sensitive and specific for IgG; however, it is now performed only in specialized laboratories due to concerns about biohazards. Indirect fluorescent antibody tests and agglutination tests, including the IgM/IgA immunosorbent agglutination assay (ISAGA), can also detect *Toxoplasma*-specific antibodies. In general, a comprehensive serologic evaluation can determine if infection is likely recent. Still, it cannot, by itself, confirm that a given ocular lesion is caused by toxoplasmosis, since a large proportion of the population may have positive antibodies from a past infection.

#### Intraocular fluid analysis

When the clinical diagnosis is uncertain or atypical, analysis of aqueous humor or vitreous fluid can provide direct evidence of ocular toxoplasmosis. Two principal approaches are used: detection of *T. gondii* DNA by PCR and detection of intraocular antibody production. PCR amplification of *T. gondii* DNA from aqueous or vitreous samples exhibits high specificity and can be highly sensitive, especially in immunocompromised patients with high parasite loads [15].

A positive PCR for *Toxoplasma* confirms the diagnosis. The reported sensitivity of ocular PCR for toxoplasmosis varies (approximately 30-50% in immunocompetent patients and up to 75% in immunosuppressed patients) [15]. Yield is highest if samples are obtained early during active retinitis (within the first 1-2 weeks of presentation) and from the vitreous cavity (closer to the site of infection). Real-time PCR techniques and targeting of multi-copy genes (like the *REP-529* element) have improved detection rates. Nonetheless, a negative PCR does not entirely rule out ocular toxoplasmosis, particularly in immunocompetent individuals with a strong inflammatory response that may suppress the organism load. In practice, PCR is most helpful in atypical cases (e.g., elderly patients with necrotizing retinitis where lymphoma is in the differential diagnosis) or in patients who are unable to mount an antibody response.

Local antibody production within the eye can be demonstrated by the Goldmann-Witmer coefficient (GWC) analysis or by immunoblotting. The GWC compares the ratio of *Toxoplasma*-specific IgG to total IgG in aqueous humor versus serum. A ratio significantly greater than 1, commonly more than two or more than three, is used as a threshold, indicating intraocular synthesis of specific antibodies, which supports an active intraocular toxoplasmosis infection rather than passive leakage of serum antibodies [16]. The sensitivity of the GWC is moderate (around 50-70%), and it may be lower early in the disease course or in immunocompromised patients who cannot produce antibodies locally. An alternative method is Western blot analysis of paired serum and aqueous samples, which can detect unique *Toxoplasma*-specific IgG bands in the aqueous.

Immunoblotting has been reported to have higher specificity than GWC and can remain reliable even when the blood-ocular barrier is disrupted [16]. In one study, combining PCR with GWC and Western blot increased the overall diagnostic sensitivity to over 90% [16]. In summary, when clinical features are not definitive, a combination of PCR (to detect parasite DNA) and intraocular antibody testing can confirm the diagnosis of ocular toxoplasmosis in most cases. It is essential to pursue these tests if an atypical presentation raises concerns for other entities, such as viral retinitis, tuberculosis, or intraocular lymphoma, since the management of those conditions differs markedly.

#### Differential diagnosis

The fundoscopic appearance of toxoplasmic retinochoroiditis can be mimicked by several other diseases. Diffuse unilateral subacute neuroretinitis (DUSN), ocular toxocariasis, and acute retinal necrosis (also known as herpetic viral retinitis) may all cause focal white retinal lesions accompanied by inflammation. Multifocal choroiditis and the acute posterior multifocal placoid pigment epitheliopathy (APMPPE) are non-infectious inflammatory syndromes that can resemble the multifocal variant of toxoplasmosis. Syphilitic punctate retinitis and tubercular chorioretinitis are important infectious considerations in persistent, unexplained cases of retinitis. In an older patient, primary vitreoretinal lymphoma (previously known as ocular reticulum cell sarcoma) is a critical masquerader to consider; lymphoma can cause subretinal infiltrates and vitritis that respond transiently to steroids, and it may coexist with positive toxoplasmosis serology, leading to potential misdiagnosis. In such cases, a diagnostic vitreous biopsy for cytology and flow cytometry is warranted to distinguish between infectious retinitis and malignancy. In practice, a careful history and examination will narrow the differential. For example, the presence of an old scar adjacent to an active lesion strongly favors toxoplasmosis. Ancillary tests (QuantiFERON-TB, VDRL/FTA-ABS for syphilis, serum ACE for sarcoid, etc.) can help exclude other uveitic etiologies in ambiguous cases [2]. Ultimately, response to therapy can also be informative - toxoplasmosis lesions typically show substantial improvement within 1-2 weeks of appropriate antiparasitic treatment, whereas a masquerade syndrome like lymphoma would not.

#### Imaging studies

Ocular imaging technologies, while not specific to toxoplasmosis, provide valuable information for assessing the extent of damage and monitoring treatment response.

#### Color fundus photography

It is helpful to document the appearance and location of lesions and scars. Sequential fundus photos allow clinicians to track the healing of an active lesion (e.g., noting the development of pigment at the borders or contraction of the lesion over time) and to detect subtle new satellite lesions on follow-up.

#### Fluorescein angiography (FA)

It can help evaluate the activity of retinochoroidal lesions and associated vasculitis. Active toxoplasmic lesions often show early hypofluorescence (blocked fluorescence due to retinal infiltrates) with late intense hyperfluorescent leakage on FA, as the lesion and surrounding inflamed tissue leak dye. This “wreath-like” late fluorescence highlights the full area of active retinitis, which may be larger than it appears clinically. Perilesional vasculitis is also well-demonstrated by FA, characterized by vessel wall staining and leakage, which sometimes extends beyond the visible borders of the lesion. Fluorescein angiography is additionally helpful in identifying complications such as retinal capillary nonperfusion or choroidal neovascular membranes adjacent to scars (which would exhibit classic leakage patterns).

#### Indocyanine green angiography (ICGA)

It can complement FA by visualizing choroidal involvement. On ICGA, active chorioretinal lesions often appear as hypofluorescent spots (indicating choriocapillaris non-perfusion) that may be more numerous or extensive than the clinically observed retinal lesions, suggesting subclinical choroidal foci. ICGA is especially helpful in differentiating toxoplasmosis from white-dot syndromes; for example, in APMPPE, the ICGA abnormalities typically far outnumber the funduscopic lesions, whereas in ocular toxoplasmosis, the ICGA hypofluorescent spots correspond more directly to the retinal lesions [8].

#### Optical coherence tomography (OCT)

It has become an indispensable tool in the evaluation of posterior uveitis, including ocular toxoplasmosis. During active toxoplasmic retinitis, OCT often reveals hyperreflective infiltration of the inner retinal layers at the site of the lesion, accompanied by full-thickness retinal disorganization. The adjacent vitreous may contain hyperreflective clumps representing inflammatory cells (sometimes termed “vitreous spots” or “hyaloid bodies”). OCT is highly sensitive in detecting even subtle epiretinal membranes or vitreoretinal traction near a healed lesion, which may not be apparent on examination but can impact vision. It can also quantify retinal thickness and edema. In eyes with macular involvement, OCT is crucial for monitoring the development or resolution of cystoid macular edema and for detecting complications, such as macular hole formation. Moreover, OCT helps distinguish old inactive scars (which exhibit a thinned, atrophic retina with possible RPE hyperplasia underneath) from new active lesions (which display swelling and tissue disruption). This can be particularly useful if a patient with known scars presents with visual changes. One needs to determine if there is a reactivation or some other cause.

#### Optical coherence tomography angiography (OCTA)

It is a newer imaging modality that can noninvasively visualize the retinal and choroidal microvasculature. In ocular toxoplasmosis, OCTA studies have demonstrated areas of capillary dropout and flow void corresponding to active lesions, as well as the presence of choroidal neovascular networks beneath some healed scars [8]. OCTA may prove beneficial in the follow-up of patients with toxoplasmic scars who have developed secondary CNV, as it can monitor the neovascular complex without repeated dye injections.

#### Ultrasound (B-scan ultrasonography)

It is another essential imaging adjunct, particularly when media opacity from vitritis, cataract, or hemorrhage obscures the view of the fundus. B-scan ultrasound can confirm the presence of dense vitritis and help rule out retinal detachment or mass lesions in eyes with poor visual acuity. In ocular toxoplasmosis, ultrasound is helpful to ensure that an unusually severe presentation with vitreous opacities is not a masquerade such as intraocular lymphoma or endophthalmitis presenting with vitreous debris. It can also detect associated findings, such as posterior hyaloid thickening, traction bands, or retinal tears, that may not be visible clinically due to haze. In summary, multimodal imaging (fundus photography, FA/ICGA, OCT) plays a supportive role in managing ocular toxoplasmosis by documenting lesions, guiding treatment decisions (e.g., starting anti-VEGF for a CNV detected on FA/OCT), and monitoring structural outcomes. Imaging is typically used in conjunction with, rather than in place of, clinical examination.

### Treatment and management

The management of ocular toxoplasmosis aims to eliminate active infection, reduce ocular inflammation, and prevent sight-threatening complications. In immunocompetent patients with small peripheral lesions and mild symptoms, there is some debate about whether treatment is always required, as such lesions may heal spontaneously over 1-2 months. However, treatment is generally recommended for all active cases, except perhaps the very most minor peripheral lesions, because therapy has been shown to accelerate lesion resolution and, more importantly, to limit the extent of retinal damage [16]. Immediate treatment is indicated for any lesion threatening the optic disc or macula, for severe vitreous inflammation, for lesions in an only-seeing eye, and in immunocompromised patients. The classic therapy for ocular toxoplasmosis was established in the 1950s and, with some modifications, remains in use today.

### First-Line (conventional) therapy

The traditional first-line regimen consists of a combination of oral pyrimethamine and sulfadiazine, along with folinic acid (to prevent pyrimethamine-induced bone marrow suppression) and systemic corticosteroids. Pyrimethamine (an antiparasitic that inhibits folate metabolism in *T. gondii*) is typically given as a loading dose of 50-100 mg, then 25-50 mg daily. Sulfadiazine (a sulfonamide antibiotic) is offered at 1-1.5 g four times daily. Leucovorin (folinic acid) 10-15 mg is administered 2-3 times weekly during this therapy to protect against pyrimethamine toxicity. Prednisone is typically initiated 1-3 days after the antimicrobials (to prevent suppressing the host response before the parasites are adequately targeted) at a dose of approximately 20-40 mg daily, with a tapering schedule of 2-6 weeks, depending on the response. This regimen is often referred to as “classic triple therapy” (pyrimethamine, sulfadiazine, steroid, with folinic acid as an adjunct). It has proven efficacy in reducing lesion size and speeding the resolution of active retinitis [16]. A prospective study by Rothova et al. found that patients undergoing antiparasitic treatment exhibited a greater reduction in lesion size and fewer recurrences in the early months compared to untreated controls with peripheral lesions [16]. Notably, that study also indicated that treatment did not significantly shorten the duration of active disease in peripheral lesions (both treated and untreated lesions healed in ~6 weeks on average), highlighting that the primary benefit of therapy is in limiting tissue destruction rather than accelerating time to healing.

An alternative first-line regimen widely used today is trimethoprim-sulfamethoxazole (TMP-SMX), administered as one double-strength tablet (160 mg trimethoprim/800 mg sulfamethoxazole) twice daily [17]. TMP-SMX has emerged as an effective and convenient therapy for ocular toxoplasmosis, with several studies demonstrating its non-inferiority to the pyrimethamine-based regimen in terms of lesion resolution and visual outcome [17]. For instance, a randomized trial showed that a 6-week course of TMP-SMX combined with steroids achieved a similar reduction in lesion size and inflammation as the classic pyrimethamine/sulfadiazine regimen, with the added advantages of fewer side effects and easier administration (oral tablets without the need for lab monitoring of blood counts as frequently) [17]. As a result, many clinicians now consider TMP-SMX, combined with a corticosteroid, as a first-line treatment, especially in settings where pyrimethamine is not readily available.

Another commonly used regimen is pyrimethamine + clindamycin (300 mg of clindamycin four times daily, with pyrimethamine and folinic acid as above).

This combination is often chosen for patients who are unable to tolerate sulfonamides. It is sometimes added as a “quadruple therapy”, where clindamycin is added to pyrimethamine, sulfadiazine, and steroid for more severe cases. One retrospective study suggested that adding clindamycin could improve intraocular inflammation and visual outcomes in certain patients [18]. However, a comparative trial by Rothova et al. indicated that adding clindamycin did not significantly enhance lesion shrinkage compared to the classic dual therapy and was associated with more side effects (notably diarrhea and the risk of pseudomembranous colitis from C. difficile) [16]. If clindamycin is used, patients must be counseled to report any severe diarrhea, and the drug should be discontinued at the first sign of colitis.

Adjunctive corticosteroid therapy is a key component of treatment in immunocompetent patients, as it dampens the immune-mediated damage that occurs in response to the dying parasites. The timing and dosage of steroids can vary. A common approach is to start oral prednisone (20-40 mg/day) 48 hours after beginning antiparasitic medication, then taper it slowly over 2 to 4 weeks as the retinitis improves. In very mild cases, some clinicians will treat with antiparasitics without steroids, but in most cases, the amount of inflammation warrants steroid use. It is crucial never to use corticosteroids without concurrent anti-toxoplasma therapy, as immunosuppression in the presence of an active infection can lead to uncontrolled parasite proliferation and devastating necrosis [14].

Topical corticosteroid drops are often used in conjunction with systemic therapy if there is significant anterior uveitis. Cycloplegic drops (e.g., atropine or cyclopentolate) are administered to prevent synechiae and reduce ciliary spasm. In immunocompromised patients, systemic steroids are used more cautiously or avoided altogether, as controlling the infection is the primary concern, given their already weakened immune systems. If used, it is typically at lower doses and only after antiparasitic treatment has begun to turn the tide of the infection.

### Alternative therapeutic approaches in sulfonamide-allergic patients

Sulfonamide antibiotics (such as sulfadiazine and the sulfa component of TMP-SMX) are a mainstay of therapy for ocular toxoplasmosis, but up to 3-8% of the general population has a hypersensitivity to sulfonamides [15]. These reactions range from mild skin rashes to severe, life-threatening conditions like Stevens-Johnson syndrome (SJS) and toxic epidermal necrolysis. In patients with a confirmed sulfonamide allergy, the traditional regimens containing sulfadiazine or sulfa-based drugs must be avoided. Fortunately, several effective alternative treatments have been documented. These include:

- Pyrimethamine + Clindamycin: This combination (with folinic acid and steroids as in standard therapy) is a well-established first choice for sulfa-allergic patients. Clindamycin at 300 mg four times daily has excellent intraocular penetration and anti-*Toxoplasma* activity. Studies and clinical experience have shown that pyrimethamine-clindamycin is as effective as pyrimethamine-sulfadiazine in resolving toxoplasmic lesions [16,19,20]. Gastrointestinal upset (especially diarrhea) is the main side effect; patients should be monitored for signs of colitis.
- Azithromycin-Based Therapy: Azithromycin, a macrolide antibiotic, has been used in combination with pyrimethamine as another alternative. A prospective trial demonstrated that pyrimethamine plus azithromycin (typically azithromycin 500 mg on day 1, followed by 250 mg daily) was equivalent in efficacy to pyrimethamine plus sulfadiazine for treating sight-threatening toxoplasmic retinochoroiditis. In contrast, azithromycin was better tolerated, with fewer adverse effects [17]. This regimen can be considered in patients who cannot use sulfa drugs. Some clinicians even use high-dose azithromycin (e.g., 1 g once weekly) with or without pyrimethamine in select cases. However, evidence supports using it in combination with antiparasitic therapy rather than monotherapy [19,20].
- Atovaquone: Atovaquone is a hydroxynaphthoquinone antiparasitic that has activity against *T. gondii*. It can be given at a dose of 750 mg four times daily. Atovaquone has the advantage of not causing the hematologic toxicity associated with pyrimethamine and not being a sulfa drug. Small studies have shown that atovaquone, either alone or combined with pyrimethamine, can successfully treat ocular toxoplasmosis lesions [8]. It is generally well-tolerated, though it is expensive and requires intake with fatty food to enhance absorption. Atovaquone is often reserved for patients who are intolerant of both sulfonamides and clindamycin, or who have contraindications to standard therapy (for instance, a pregnant patient in the first trimester with a sulfa allergy-pyrimethamine is typically avoided in first trimester due to teratogenic potential, so atovaquone or spiramycin may be options in that scenario).
- Trimethoprim-Sulfamethoxazole (Desensitization or Partial Allergy): If the sulfonamide allergy is mild (e.g., a non-serious rash) or not definitively established, a carefully monitored challenge or desensitization protocol with TMP-SMX can be attempted. In some cases, patients with a history of sulfa allergy have been able to tolerate TMP-SMX when given gradually increasing doses under supervision [15]. This approach should only be undertaken by experienced specialists, as severe reactions can recur. If successful, the patient can then receive the highly effective TMP-SMX regimen. Desensitization is generally contraindicated if the prior response was severe (Stevens-Johnson syndrome or organ involvement).
- Intravitreal Therapy: For patients who cannot take systemic antiparasitic therapy (due to allergies or systemic contraindications), intravitreal injection of antimicrobial agents offers a way to treat the eye locally. The most commonly used protocol involves the injection of intravitreal clindamycin (1.0 mg/0.1 ml) combined with intravitreal dexamethasone (0.4 mg/0.1 ml) into the vitreous cavity, typically administered as one or two injections a few weeks apart. This delivers high drug concentrations to the retina without systemic exposure. A randomized trial involving 68 patients found that intravitreal clindamycin and dexamethasone therapy was non-inferior to classic oral therapy in terms of lesion size reduction and visual acuity outcomes at 6 weeks [18]. Notably, in that study, the difference in efficacy between intravitreal and systemic treatment was influenced by IgM serology status: patients who were IgM-positive (suggesting recently acquired infection) responded better to systemic therapy, whereas those who were IgM-negative had equally good outcomes with intravitreal treatment [18]. Intravitreal therapy can be beneficial in sulfa-allergic patients or others who cannot use systemic treatment. Its advantages include avoiding systemic side effects and eliminating the need for patient compliance with a long oral regimen. However, intravitreal treatment addresses only the eye; it does not treat systemic infection (which could be relevant in congenital cases or immunocompromised patients), and it carries risks inherent to any intraocular injection (such as endophthalmitis or retinal detachment, though these are rare when done correctly). Intravitreal injections also do not prevent new lesions from occurring elsewhere in the retina later, since there is no sustained systemic suppression of the parasite. Therefore, some experts use intravitreal clindamycin as an adjunct to reduce reliance on systemic therapy rather than a complete replacement, or solely for cases where systemic therapy is not an option [19,20].

In summary, for patients with sulfonamide allergies, there are several effective therapeutic strategies to manage ocular toxoplasmosis without using sulfa drugs. Pyrimethamine plus clindamycin is a proven regimen in this scenario, and pyrimethamine plus azithromycin is another evidence-based alternative. Atovaquone offers a non-sulfonamide option, albeit with cost considerations. Intravitreal antimicrobial injections provide a targeted approach that bypasses systemic exposure entirely.

The choice among these alternatives should be individualized based on the patient’s specific allergies, the severity and location of the retinal lesions, and the patient’s overall health status. In all cases, close monitoring is required. Adjunctive corticosteroid therapy (systemic or local) is usually still indicated in sulfa-allergic patients receiving alternative antimicrobial regimens, unless contraindicated, to control inflammation.

It is also worth emphasizing that any patient who has experienced a severe sulfonamide reaction (such as SJS) should avoid not only sulfa antibiotics but also consider avoidance of other sulfonamide-containing medications when possible, and this should be documented in their medical record.

### Adjunctive and supportive therapies

Regardless of the antimicrobial regimen chosen, all patients with ocular toxoplasmosis benefit from measures to control inflammation and support ocular health. Topical corticosteroid and cycloplegic eye drops are used if anterior uveitis is present to relieve pain and prevent synechiae. In cases of elevated intraocular pressure (IOP), ocular hypotensive medications (such as topical β-blockers, α-agonists, or carbonic anhydrase inhibitors) are administered to protect the optic nerve. Patients are followed frequently (every 1-2 weeks in the early phase) to ensure the lesion is responding to treatment. A reduction in vitreous haze and the beginnings of scar formation at the lesion edges indicate improvement. Suppose there is inadequate response or lesion enlargement after 2 weeks of compliant therapy. In that case, one should re-evaluate the diagnosis or consider intensifying treatment (for example, adding a second agent or switching regimens).

Treatment is generally continued for at least 4-6 weeks, and often longer (up to 2-3 months) if the lesion is large or located in the posterior pole, to ensure complete resolution of active retinitis.

After successful treatment of an episode, patients with ocular toxoplasmosis should be educated about the potential for recurrence. Approximately 40-50% of patients will experience one or more recurrent episodes in their lifetime, often at the border of an old scar or in a different location in the same eye [1]. The highest risk of recurrence is within the first 1-2 years after an episode. In frequent recurrences, some clinicians prescribe chronic suppressive therapy (for example, one TMP-SMX double-strength tablet three times weekly) to reduce relapse rates. A randomized trial demonstrated that such low-dose TMP-SMX prophylaxis can significantly decrease the risk of toxoplasmosis recurrence while the patient is on therapy [19]. This approach is primarily considered in immunocompromised patients or those with sight-threatening lesions in the only eye. In any case, regular ophthalmic follow-up is advisable.

Patients should be instructed to return promptly if they notice new floaters or changes in their vision, as early re-treatment of a recurrence can help prevent additional retinal damage.

## Conclusion

Ocular toxoplasmosis remains a challenging yet treatable cause of uveitis and vision loss. It is a prevalent infection worldwide and a significant public health concern in endemic regions. The diagnosis is primarily clinical, based on the recognition of characteristic necrotizing retinitis; however, adjunctive laboratory tests, such as PCR analysis of intraocular fluid and local antibody analysis, can be invaluable, particularly in atypical cases. Standard therapy, which involves combinations of antiparasitic drugs and corticosteroids, has been the cornerstone of management for decades and has a strong track record of efficacy in controlling active disease and preserving vision. At the same time, recent developments—including simplified antibiotic regimens (such as TMP-SMX), the viability of intravitreal therapy, and preventive strategies to reduce recurrences—have expanded our therapeutic options.

Patients with sulfonamide allergies, who previously posed a complex management problem, can now be treated effectively with alternative systemic medications or localized therapy without compromising outcomes.

Ultimately, the goal of therapy is not only to heal the current lesion but also to minimize permanent visual impairment. Timely intervention can limit retinochoroidal scarring and its sequelae. In refractory or severe cases, a multidisciplinary approach involving uveitis specialists, retina surgeons, and, when needed, systemic disease specialists (e.g., infectious disease or immunology for immunocompromised hosts) is recommended. Further research is warranted to refine treatment duration, develop anti-parasitic drugs with improved safety profiles, and explore adjunct therapies (such as anti-inflammatory or immunomodulatory agents) that could reduce retinal damage. Additionally, a better understanding of immunopathology, for instance, the role of cytokines such as IL-17 and regulatory T cells, may open doors to targeted therapies that modulate the host response for improved outcomes. Ongoing and future studies will ideally lead to evidence-based consensus on questions like when to initiate prophylactic treatment and how to manage special populations (e.g., pregnant women, pediatric cases). In conclusion, with vigilant clinical care and use of the expanding range of therapeutic options, the prognosis for patients with ocular toxoplasmosis, even those who cannot tolerate first-line medications, has significantly improved. Continued efforts in research and clinical practice are necessary to reduce the burden of this disease further and prevent vision loss in affected individuals.
